# Genetic Testing Confirmed the Early Diagnosis of X-Linked Hypophosphatemic Rickets in a 7-Month-Old Infant

**DOI:** 10.1177/2324709615598167

**Published:** 2015-08-03

**Authors:** Kok Siong Poon, Andrew Anjian Sng, Cindy Weili Ho, Evelyn Siew-Chuan Koay, Kah Yin Loke

**Affiliations:** 1 National University Health System, Singapore; 2 National University of Singapore, Singapore

**Keywords:** X-linked hypophosphatemic rickets, *PHEX* gene, splice-site mutation, genetic testing

## Abstract

Loss-of-function mutations in the *p*hosphate regulating gene with *h*omologies to *e*ndopeptidases on the *X*-chromosome (*PHEX*) have been causally associated with X-linked hypophosphatemic rickets (XLHR). The early diagnosis of XLHR in infants is challenging when it is based solely on clinical features and biochemical findings. We report a 7-month-old boy with a family history of hypophosphatemic rickets., who demonstrated early clinical evidence of rickets, although serial biochemical findings could not definitively confirm rickets. A sequencing assay targeting the *PHEX* gene was first performed on the mother’s DNA to screen for mutations in the 5′UTR, 22 coding exons, and the exon-intron junctions. Targeted mutation analysis and mRNA studies were subsequently performed on the boys’ DNA to investigate the pathogenicity of the identified mutation. Genetic screening of the *PHEX* gene revealed a novel mutation, c.1080-2A>C, at the splice acceptor site in intron 9. The detection of an aberrant mRNA transcript with skipped (loss of) exon 10 establishes its pathogenicity and confirms the diagnosis of XLHR in this infant. Genetic testing of the *PHEX* gene resulted in early diagnosis of XLHR, thus enabling initiation of therapy and prevention of progressive rachitic changes in the infant.

## Introduction

X-linked dominant hypophosphatemic rickets (XLHR) was first described in 1937^1^ and it is a rare genetic disorder with an incidence of 1 in 20 000.^2^ In addition to X-linked inheritance, hypophosphatemic rickets demonstrates autosomal dominant and autosomal recessive inheritance modes in which the clinical phenotype can be identical. As a consequence, abnormalities in at least 9 genes have been reported to be associated with all forms of hypophosphatemic rickets, either with heritable transmissions or in sporadic cases.^3^ The clinical diagnostic approach is based on the clinical phenotype supported by screening of the implicated genes in hypophosphatemic rickets. Loss-of-function mutations in the *p*hosphate regulating gene with *h*omologies to *e*ndopeptidases on the *X*-chromosome (*PHEX*) have been causally associated with XLHR. The *PHEX* gene is located on the X chromosome, at Xp22.1-22.2 and spans 225 kb of genomic sequence.^4,5^ The 6.6-kb mRNA transcript consists of 22 exons that encode 749 amino acids.^6^ More than 300 different mutations have been reported in the *PHEX* Locus Database, PHEXdb (http://www.phexdb.mcgill.ca; accessed on April 9, 2015).^7^ Germ-line mutations including missense, nonsense, insertion, deletion, in-del, and splice-site mutations have been described in the *PHEX* gene since its discovery in 1995. In addition to familial-specific mutations, de novo mutations were also reported in sporadic XLHR cases.^8-10^

The clinical phenotypes of XLHR include rickets, short stature, bone pain, bone deformities, and dental abnormalities.^11^ Biochemical findings in XLHR patients demonstrate low serum phosphate, low to normal serum 1,25-dihydroxyvitamin D, normal serum calcium, normal serum parathyroid hormone, and elevated alkaline phosphatase activity.^11,12^ Distinguishing XLHR from other forms of hypophosphatemic rickets in infancy is challenging, based solely on the clinical features and biochemical findings. Differential diagnosis can be established by correlating the clinical findings with accurate family history and genetic analysis. We report a familial case of XLHR of a mother and her 2 sons, with a novel splice-site mutation. Molecular genetic analysis was successfully applied to genetically diagnose XLHR, which then enabled early treatment in the 7-month-old male infant in this family.

## Case Presentation

**Figure 1.**
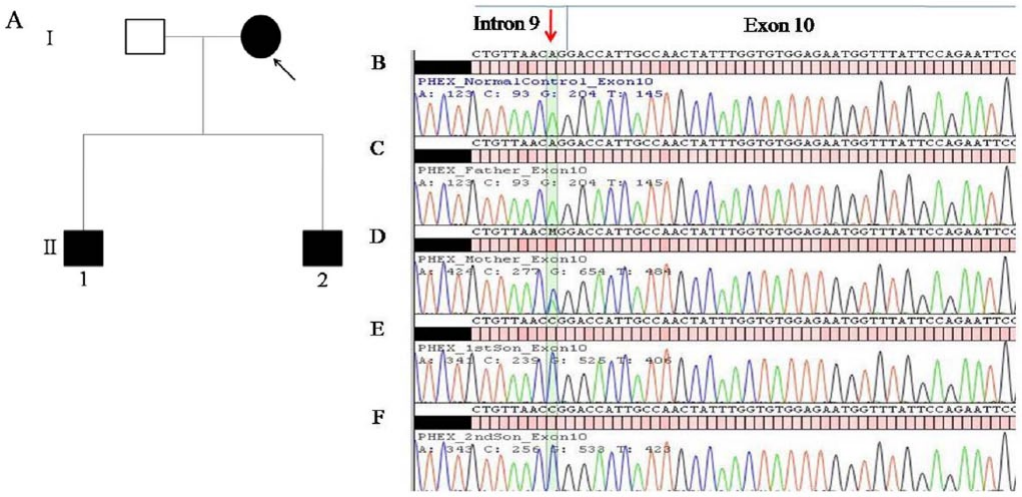
(A) Pedigree of a Chinese family with XLHR. Arrow indicates the proband. II-1 is the affected son and II-2 is the pre-symptomatic affected son. (B-F) Electropherograms showing the aligned genomic sequence of the last 10 nucleotides of the 3′ splice acceptor site of intron 9 and the first 47 nucleotides of exon 10. Nucleotide position of c.1080-2A>C mutation is indicated by an arrow. The wild-type A nucleotide was detected in a normal control (B) and in the father (C). The mutation (A>C nucleotide change) was detected in heterozygous state in the mother (D) and in hemizygous state in the 2 male offspring (E and F).

A 1-month-old boy, the second boy of a non-consanguineous marriage, was referred to the pediatric endocrine service for possible rickets, as his mother and older brother (Figure 1A) were previously diagnosed clinically to have XLHR by biochemical investigations. He was born full term by an elective lower segment caesarean section, with a birth weight of 2.93 kg. His initial serum phosphate was low at 1.43 mmol/L (reference range 1.64-2.47), serum calcium was 2.49 mmol/L (reference range 2.05-2.85), serum creatinine was 46 µmol/L (reference range 23-46), serum alkaline phosphatase level was 332 U/L (reference range 70-350), serum 25-hydroxy vitamin D level was 46.3 µg/L (>30: normal), urine phosphate was 4.2 mmol/L and urine creatinine was 0.7 mmol/L, with a normal urine phosphate to creatinine ratio of 6.0 (reference range 1.2-19). The calculated fractional excretion of phosphate (using the formula of urine phosphate/plasma phosphate × plasma creatinine/urine creatinine) was 19%. Based on this, the renal threshold for phosphate concentration was 1.18 mmol/100 mL glomerular filtration rate (GFR), which was higher than the normal range for infancy (reference range 0.15-0.34 mmol/100 mL GFR), suggesting that he was not losing phosphate in the urine.^13,14^

He was subsequently followed-up with serial monitoring of growth and his serum phosphate levels. At 3 months of age, he was noted to have very mild genu varus. His serum phosphate had decreased to 1.13 mmol/L (reference range 1.64-2.47), with a rising serum alkaline phosphatase of 434 U/L (reference range 70-350). The urine phosphate to creatinine ratio was 8.5 (reference range 1.2-19). However, his calculated renal threshold phosphate concentration was 0.92 mmol/100 mL GFR, which was still higher than the normal range for infancy, even though it had decreased from the previous level. At 5 months of age, his serum phosphate was low at 1.00 mmol/L with a normal urine phosphate to creatinine ratio of 7.6 (reference range 1.2-19). However, his renal threshold for phosphate concentration was 0.98 mmol/100 mL GFR, which was still higher than the normal range of 0.12 to 0.26 mmol/100 mL GFR, suggesting that there was still no significant renal phosphate wasting.^14^

**Figure 2.**
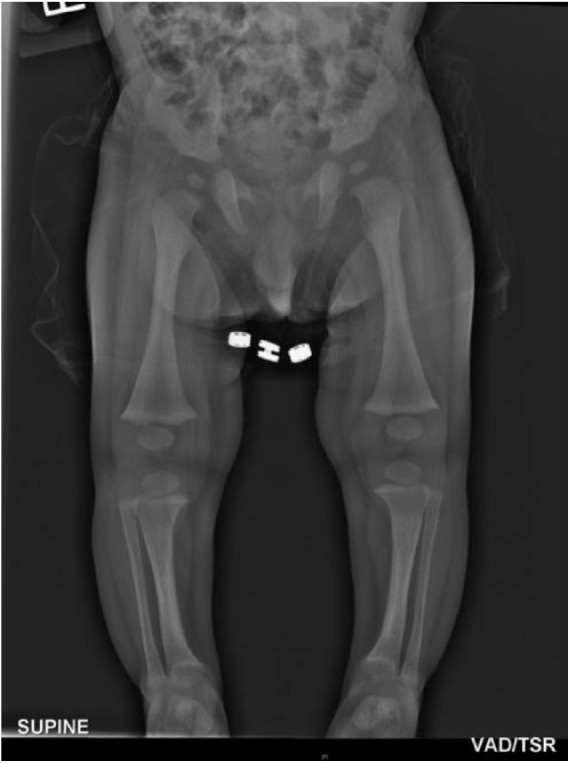
Long limb radiograph showing the features of the lower limbs of the 7-month-old infant. The long bones of the lower limbs demonstrate splaying of the metaphyses with fraying, but no widening of the epiphysis. There is also mild bowing of the tibia.

At 7-month review, his recumbent length was 69.0 cm (50th to 75th percentile), weight was 7.52 kg (25th to 50th percentile), and head circumference measured 45.2 cm (90th to 97th percentile). His anterior fontanelle was small and there was persistent mild genu varus with no flaring of the wrists or ankles. His serum phosphate measured 1.05 mmol/L (reference range 1.64-2.47), serum alkaline phosphatase measured 459 U/L (reference range 70-350), the urine phosphate to creatinine ratio was still normal at 11.7 (reference range 1.2-19), and the renal threshold for phosphate concentration was 0.84 mmol/100 mL GFR, which again suggested no renal phosphate wasting as this was above the quoted normal range for infants.^14^ The serial biochemical results are tabulated in Table 1. However, the plain radiograph of the lower limbs showed evidence of rickets with cupping and splaying of the metaphyses and bilateral genu varus (Figure 2).

**Table 1.** Serial Biochemical Results Measured During the First 7 Months of Age of the Infant.

	Age (Months)				
Biochemical Measurements	1	3	5	7	Normal Levels
Serum calcium (mmol/L)	2.57	2.52	2.38	2.55	2.05-2.85
Serum phosphate (mmol/L)	1.43	1.13	1.0	1.05	1.64-2.47
ALP (U/L)	332	434	385	459	70-350
PTH (pmol/L)	2.8	3.8	6.6	5.4	1.3-9.3
TmPO4/GFR (mmol/100 mL GFR) [normal ranges]	1.18 [0.15-0.34]	0.92 [0.148-0.33]	0.98 [0.12-0.26]	0.84 [0.12-0.26]	

Abbreviations: ALP, alkaline phosphatase; PTH, parathyroid hormone; TmPO4/GFR, renal threshold phosphate concentration. Normal reference ranges for TmPO4/GFR are from Arch Dis Child. 1986;61:677-681.

Written informed consent was then obtained for the *PHEX* gene analysis. However, since this was primarily to help the patient in diagnosis and management, ethics approval was not obtained from the institution. Genetic testing of the *PHEX* gene, which was just set up at our hospital, demonstrated a novel splice-site mutation c.1080-2A>C. The diagnosis of XHLR was secured and he was commenced on oral phosphate replacement with vitamin D.

## Molecular Genetics Investigations

### Sequence Analysis of Genomic Sequence of the PHEX Gene

Genomic DNA was extracted from 3 mL of peripheral blood collected in EDTA-anticoagulant tubes, using the Gentra Puregene Blood Kit (Qiagen, Hilden, Germany). Twenty-two sets of polymerase chain reaction (PCR) primers (Table 2) targeting part of the 5′ untranslated region (UTR), all 22 coding exons and the exon-intron junctions of the *PHEX* gene were designed using an online primer design tool, Primer3.^15,16^ Amplification by PCR was performed in a 20-µL reaction consisting of 1× HotStarTaq Plus Master Mix (Qiagen), 5 mmol/L each of the forward and reverse primers, 1× Q solution (Qiagen) and 100 ng of the extracted genomic DNA. Thermal cycling conditions include an initial denaturation at 95°C for 5 minutes, followed by 40 cycles of 95°C for 30 seconds, 61°C for 30 seconds, 72°C for 30 seconds and a final extension at 72°C for 10 minutes. The PCR products were separated by electrophoresis with 2% agarose gel and purified using QIAquick gel extraction kit (Qiagen). Bidirectional sequencing was performed using BigDye Terminator kit version 3.1 (Applied Biosystems, Austin, TX) with the respective forward and reverse PCR primers on a 3100xl Genetic Analyzer (Applied Biosystems). The Assign ATF (Conexio Genomics, Fremantle, Australia) software package was used for sequence alignments against the genomic reference sequence, NG_007563.2. Mutation screening in the 5′UTR and entire coding region of the *PHEX* gene was performed on the proband’s genomic DNA while targeted mutation analysis using primer set amplifying exon 10 and its intron-exon junctions was performed on the other family members.

**Table 2.** Sequence of Primers Used in PCR Amplification and Sanger Sequencing of the *PHEX* Gene.

Targeted *PHEX* Gene Genomic Region	Forward Primer	Reverse Primer	PCR Product Size (Base Pairs)
Partial 5′UTR and Exon 1	gaaagccaaggcaaccaata	aacagaatcagccagccact	600
Exon 2	tgtttccgagggtggtttac	gctccactgtttcacaccaa	313
Exon 3	aaggcttggaaactggttga	tgttgagatctgggagtcca	452
Exon 4	ttgaacctcatgcaacttgg	ccaccaagccagtaacaaca	465
Exon 5	ccaccccacctcttttacct	ggcagcatgagtctctttcc	461
Exon 6	gccaaagtgcctatttgcat	cctgcattgggaatatggtc	362
Exon 7	tggctggacatctctgtctg	caatgggcaatgacacaaaa	499
Exon 8	tttttctcttccccgagttg	tgagccaatgccaacaatta	503
Exon 9	tctggatggcaatgatcagga	aggatgtgagaagggaagcc	391
Exon 10	gtggtgaaaagcaagccaat	aaaactctgggggaaaatgg	599
Exon 11	gggttagggtgtgcagtgtt	gacaatacccacaggccact	355
Exon 12	cagagcatggagtcaagctg	caagccatgtgcctcttaca	520
Exon 13	atttttgcccttcacagtgg	gctacgcatcgtttctgaca	436
Exon 14	aggactcgtgagccaagaaa	ggcaagccagctactctgac	564
Exon 15	gtccaacatccccattgttc	caaccttccttcaccagcat	312
Exon 16	accagtgcaaaatggtttcc	ttccatggcttctttctgct	388
Exon 17	gcagtttatcttggctttcca	ttattgcaagccatcacagc	338
Exon 18	ttttgaaggcttgtcgaggt	ttcagcaggtatggggtagg	379
Exon 19	ttgatgcctcttgctgaatg	catgcttcgatctgatggtg	433
Exon 20	taaggcctttttgcaggtgt	ttaccacagggctgctaacc	537
Exon 21	gcactcaggggcagactact	tctggtagagcccttggatg	583
Exon 22	gtgcttggcattcaggagat	tctccaggcctaaagcaatg	400

### Sequence Analysis of PHEX mRNA Sequence

Mononuclear cells were isolated from the peripheral blood with Ficoll-Paque PLUS (GE Healthcare Bio-Sciences, Uppsala, Sweden) by gradient centrifugation. Total RNA was subsequently extracted from mononuclear cells using TRIzol Reagent (Life Technologies, Carlsbad, CA). One microgram of the extracted RNA was subjected to reverse-transcription reaction using SuperScript VILO cDNA Synthesis Kit (Life Technologies). Two microliters of the cDNA generated was subjected to PCR with a primer set (mRNA9F: acagttccagagcagagcat; mRNA12R: ttcctgcatccatccactca) to amplify a 594-bp PCR product, which spans exon 9 to exon 12 of the *PHEX* mRNA transcript. The thermal cycling and subsequent sequencing analysis procedures used were described in the previous section. Sequence alignment was based on the mRNA reference sequence, NM_000444.5.

### In Silico Splicing Analyses

Bioinformatic analyses of potential splicing defects was performed using Splice-Port (http://spliceport.cbcb.umd.edu/)^17^ and BDGP Spice Site Prediction by Neural Network (http://www.fruitfly.org/seq_tools/splice.html)^18^ to predict putative 5′ splice donor and 3′ splice acceptor sites. Potential exon-skipping and activation of cryptic splice sites were predicted using CRYP-SKIP (http://cryp-skip.img.cas.cz/).^19^ Both wild-type and mutant flanking intronic sequences of exon 10 of the *PHEX* gene were subjected to analysis using default parameters.

## Results

Direct sequencing of the mother’s *PHEX* gene revealed that she was heterozygous for a novel splice-site mutation, c.1080A>C at the conserved −2 position at the 3′ splice acceptor site in intron 9 (Figure 1D). The mutation was also identified in the hemizygous state in her 2 sons (Figure 1E, 1F), but was not detected in the healthy father (Figure 1C).

**Figure 3.**
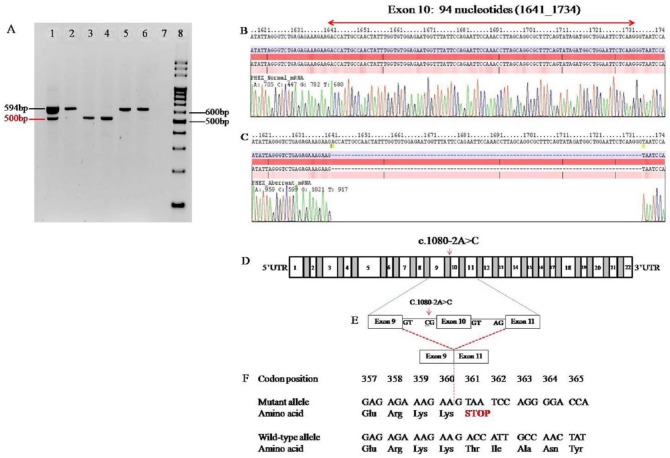
(A) Electrophoresis of RT-PCR products derived from the mother (proband), the 2 sons and the controls. Lane 1, mother; Lane 2, father; Lane 3, elder son; Lane 4, younger son; Lane 5, normal female control; Lane 6, normal male control; Lane 7, non-template control; Lane 8, DNA marker. Electropherograms showing the nucleotides of the RT-PCR products of 594 bp in (B) and 500 bp in (C) The 94-bp coding sequence of exon 10 was missing in the 500-bp RT-PCR product in (C). (D) Diagrammatic representation of the genomic region of the *PHEX* gene. Exons 1 to 22 are represented as numbered boxes while grayed boxes are intronic regions. (E) Schematic representation of aberrant mRNA transcripts. In the mutant allele with c.1080A>C mutation, the altered splice acceptor site at intron 9 causes the production of an aberrant mRNA transcript with skipped (loss of) exon 10. (F) Parts of the protein translation from the aberrant (upper) and normal (lower) mRNA transcripts are compared. Premature stop codon is introduced at codon 361 resulting in a truncated PHEX protein with 360 amino acid fragments.

In the current study, mRNA analysis was performed to interrogate the potential effects of the splice-site mutation. The results of RT-PCR reactions amplifying exons 9 to 12 from the *PHEX* mRNA demonstrated that in addition to the normal 594-bp PCR product, a shorter 500-bp PCR product was also observed in the mother’s sample (Figure 3). On the other hand, only the single shorter 500-bp PCR product was detected in her 2 sons. The missing 94-bp nucleotide segment corresponded to the size of exon 10. Sequencing results of cDNA revealed the aberrant mRNA transcript is lacking the entire coding sequences of exon 10 (Figure 3).

## Discussion

We report a 7-month-old infant with clinical evidence of early rickets that was not confirmed by serial measurements of the renal threshold for phosphate concentration, based on the reported norms for infants, which was the clinical standard for diagnosing XLHR. The renal threshold for phosphate concentration was chosen as the parameter to assess phosphate excretion, since it is independent of dietary phosphate, serum phosphate levels and variability in glomerular filtration rate, especially in neonates and young infants. Although the serum phosphate was low and even though there was circumstantial evidence that the infant was affected, it was essential to demonstrate to the parents and convince them that excessive phosphate was excreted in the urine based on the urine phosphate threshold for phosphate excretion, before their second son was committed to lifelong treatment with phosphate. Moreover, the parents were concerned about the difficulty in serving phosphate to the infant, in addition to the potential side effects of phosphate treatment including diarrhea and early nephrocalcinosis, which can develop as a consequence of intermittent episodes of hyperphosphatemia with associated hypocalcaemia and secondary hyperparathyroidism.

Genetic analysis, however, confirmed the presence of a novel *PHEX* gene mutation whose pathogenicity was demonstrated, thus providing both a genetic diagnosis and confirmation of the clinical suspicion that this infant suffered from XLHR, just like his mother and brother. The older brother was only diagnosed to have XLHR at the age of 11 months, based on the renal threshold for phosphate concentration. The overlap between the reference range for this biochemical parameter in normal infants and those with XLHR did not allow us to make a definitive diagnosis of XLHR in early infancy. Genetic screening provides a more sensitive and accurate method of early diagnosis, enabling early treatment and reducing the risk of progressive deformity.

In XLHR, approximately 23% of mutations detected in the *PHEX* gene were reported as splice-site mutations in the PHEXdb.^7^ Another recent study also reported a similar frequency of splice-site mutations in a large cohort of hypophosphatemic patients from 118 pedigrees.^8^ The novel c.1080A>C splice site mutation identified in this pedigree has not been previously reported, although another closely related splice-site mutation, c.1080A>G, has been reported at the same nucleotide position.^5^ The c.1080A>G mutation was described to potentially result in complete skipping of exon 10, followed by a frameshift of the mis-spliced exon sequences.

Both Splice-Port and BDGP Spice Site Prediction by Neural Network correctly predicted the canonical 3′ splice acceptor site at intron 9 of wild-type *PHEX* gene. Analysis by CRYP-SKIP predicted that the splice-site mutation will favor skipping of exon 10 with a very low probability value of cryptic splice-site activation (*P*CR-E), 0.07. These data suggest that the novel mutation would have a negative impact on the *PHEX* mRNA splicing. To assess the functional effects of this splice-site mutation, mRNA analysis was performed on total RNA extracted from peripheral blood mononuclear cells, in order to overcome the difficulties in accessing the tissues including osteoblasts, osteocytes, and odontoblasts, which have abundant expression of PHEX protein.^20,21^ The PHEX protein has significant structural homology to members of the type II integral membrane zinc-dependent endopeptidase family, with short N-terminal cytoplasmic domains, a single transmembrane domain and a long extracellular domain.^2^ Based on sequencing data, the aberrant mRNA transcript with complete skipping of exon 10 is predicted to have normal amino acid sequences for the first 360 codons followed by a premature termination codon, UAA at codon 361 (Figure 3). This resultant protein would lack the amino acids 361 to 749, which is the major portion of the extracellular domain with the entire zinc-binding motif. The normal PHEX protein has 10 conserved cysteine residues.^2^ The truncated protein loses 5 cysteine residues, which are likely involved in disulfide bond formation and is expected to cause a defective secondary protein structure and enzymatic activity.^10^ The detection of the aberrant mRNA transcript and the deduced loss-of-function PHEX protein provides evidence for the pathogenic role of this novel c.1080-2A>C splice-site mutation in causing the XLHR.

## Conclusion

In conclusion, we report a novel mutation, c.1080-2A>C, at the splice acceptor site of intron 9 of the *PHEX* gene and confirmed its pathogenicity in altering gene transcription by the detection of an aberrant mRNA transcript with a skipped exon 10. Targeted molecular testing allowed for early diagnosis of XLHR in this 7-month-old male infant, thus enabling prompt initiation of treatment to reduce and halt the development of rachitic changes, which may otherwise have further compromised the development and growth of the affected child.
